# Influence of prior probability information on large language model performance in radiological diagnosis

**DOI:** 10.1007/s11604-025-01743-3

**Published:** 2025-02-05

**Authors:** Takahiro Fukushima, Ryo Kurokawa, Akifumi Hagiwara, Yuki Sonoda, Yusuke Asari, Mariko Kurokawa, Jun Kanzawa, Wataru Gonoi, Osamu Abe

**Affiliations:** Department of Radiology, Graduate School of Medicine, The University of Tokyo, 7-3-1 Hongo, Bunkyo-Ku, Tokyo, 113-8655 Japan

**Keywords:** Large language model, Artificial intelligence, Claude 3.5 Sonnet, Bayes’ theorem

## Abstract

**Purpose:**

Large language models (LLMs) show promise in radiological diagnosis, but their performance may be affected by the context of the cases presented. Our purpose is to investigate how providing information about prior probabilities influences the diagnostic performance of an LLM in radiological quiz cases.

**Materials and methods:**

We analyzed 322 consecutive cases from Radiology’s “Diagnosis Please” quiz using Claude 3.5 Sonnet under three conditions: without context (Condition 1), informed as quiz cases (Condition 2), and presented as primary care cases (Condition 3). Diagnostic accuracy was compared using McNemar’s test.

**Results:**

The overall accuracy rate significantly improved in Condition 2 compared to Condition 1 (70.2% vs. 64.9%, *p* = 0.029). Conversely, the accuracy rate significantly decreased in Condition 3 compared to Condition 1 (59.9% vs. 64.9%, *p* = 0.027).

**Conclusions:**

Providing information that may influence prior probabilities significantly affects the diagnostic performance of the LLM in radiological cases. This suggests that LLMs may incorporate Bayesian-like principles and adjust the weighting of their diagnostic responses based on prior information, highlighting the potential for optimizing LLM’s performance in clinical settings by providing relevant contextual information.

## Introduction

Large language models (LLMs) are neural network models trained on huge amounts of text data and have shown excellent performance in natural language processing tasks. They are used in a variety of fields, including medicine, and attempts to utilize LLMs in radiological diagnosis have begun as follows.

In a study by Ueda et al. [1], GPT-4 (OpenAI, San Francisco, USA) achieved over 50% accuracy rate, correctly answering 170 out of 313 cases of various disease categories in Radiology’s “Diagnosis Please” quiz cases based on the patient’s history and the imaging findings. Similarly, Horiuchi et al. [2] reported that the GPT-4 achieved a diagnostic accuracy of 50% (50/100 cases) in the American Journal of Neuroradiology’s “Case of the Week” quiz series of neuroradiology cases. Furthermore, recent studies have demonstrated a significant improvement in the diagnostic capabilities of LLMs when provided with key images in addition to textual information [3]. Comparisons have been made both within and between vendors [4, 5] as well as between LLMs and human radiologists [6]. While current LLMs do not yet match the diagnostic accuracy of human radiologists, they have shown potential as powerful supportive tools.

To enhance LLM diagnostic performance and facilitate future clinical applications, it is crucial to accurately evaluate their capabilities and understand their characteristics. Previous studies have utilized case collections well-suited for research, such as “Diagnosis Please” cases in Radiology, which provide comprehensive clinical histories, radiological images, and confirmed final diagnoses (logically deducible).

However, it is important to emphasize that the above studies have consistently placed LLMs at a disadvantage by withholding information about prior probabilities—an unwritten rule that human radiologists inherently consider. Quiz cases and real clinical scenarios differ substantially in terms of prior probabilities, which are vital contributors to diagnosis. Human radiologists understand that expert-level quiz cases are more likely to feature rare diseases, uncommon presentations of common diseases, educational mimickers, or recently discovered conditions, rather than typical presentations of common diseases, such as cerebral infarction or subarachnoid hemorrhage. In real-world clinical settings, disease prevalence varies between primary care clinics and tertiary referral hospitals [7], and human radiologists can judge which answers are more likely to be correct based on institutional and regional characteristics, thereby improving their diagnostic accuracy [8, 9].

The importance of prior probabilities in determining the final diagnosis is supported by Bayes’ theorem [8]. Bayes’ theorem represents the probabilistic nature of diagnostic reasoning in a mathematical formula and is generally expressed in the context of clinical reasoning using posterior probabilities (predictive values), sensitivity (Sn) and specificity (Sp), and prior probabilities (often called prevalence; Prev) as follows [8]:

$$\text{Positive Predictive Value }\left(\text{PPV}\right)=\frac{\text{Sn}\times \text{Prev}}{\left[\text{Sn}\times \text{Prev}+\left(1-\text{Sp}\right)\times \left(1-\text{Prev}\right)\right]}$$

$$\text{Negative Predictive Value }\left(\text{NPV}\right)=\frac{\text{Sp}\times \left(1-\text{Prev}\right)}{\left[\left(1-\text{Sn}\right)\times \text{Prev}+\text{Sp}\times \left(1-\text{Prev}\right)\right]}$$

This equation represents the relationship between probabilities acting in the diagnostic process, demonstrating that the critical determinants of posterior probabilities for a disease are the prior probabilities and test characteristics. The Bayesian approach has been known to be useful for diagnosing rare diseases; as new evidence emerges, clinicians can update their prior probabilities and identify rare diseases that initially seemed unlikely [10].

We hypothesized that Bayes’ theorem might play a crucial role not only in human diagnosis but also in the diagnostic process of state-of-the-art LLMs. Specifically, we predicted that providing information as prompts to assist in estimating prior probabilities would enhance the diagnostic performance of Claude 3.5 Sonnet. Conversely, we anticipated that setting a context where encountering cases likely to be the correct answers in quizzes is infrequent would decrease diagnostic performance. To the best of our knowledge, no previous study has compared multiple factors—including false information—that influence prior probability through prompt engineering, as done in this study. We believe this represents a novel approach. In a previous study, Sonoda et al. [11] used diagnosis please cases and demonstrated that structured clinical reasoning prompts can enhance the diagnostic accuracy of LLM. Although this study uses the same LLM model and data set as the previous study, and they are similar in that they examine differences in diagnostic performance between methods, they are very different in terms of their content. They differ substantially in that the previous study focused on differences in the way the information was presented, whereas the present study modified the information itself that was given, so these two studies should be discussed separately.

The aim of this study is to prove our hypothesis that Bayes’ theorem is important for LLMs and that giving them information which may change prior probabilities will affect their diagnostic ability, and to further elucidate the characteristics of LLMs in medical diagnosis.

## Materials and methods

Ethical approval was not required as this study exclusively used data from previously published articles.

We used clinical history and author-provided figure legends of 322 consecutive cases (from Aug 1 1998 to Oct 31 2023) from Diagnosis Please, a monthly quiz case collection for diagnostic imaging physicians published in Radiology.

We used Claude 3.5 Sonnet (Anthropic, San Francisco, United States; released on June 27, 2024) to list the primary diagnoses and two differential diagnoses for the cases. Application programming interfaces were used to access the model (Claude 3.5 Sonnet: claude-3-5-sonnet-20240620) on August 17, 2024. To ensure reproducibility, we specified 0.0 as the temperature parameter. Temperature is a variable that controls the randomness of the LLM’s output; the closer to 0, the closer the LLM presents realistic answers with fewer creative elements. To avoid the influence of in-context learning [12], each evaluation was performed in an independent session.

We used three different prompts as follows:

- Condition 1: not given as being a quiz case and no situation is set: “Assuming you are a physician, please respond with the most likely diagnosis and the next two most likely differential diagnoses based on the attached information.”
- Condition 2: given as being quiz cases: The prompt for Condition 1 with “This is a quiz case for diagnostic radiologists, and your goal is to correctly answer this quiz case. In this quiz case, diseases with concepts established within the last 5 years, rare diseases, rare presentations of common diseases, and educational mimickers are more likely to be asked, while typical presentations of common diseases are less likely to be questioned.”
- Condition 3: presented as a primary care situation: “You are an experienced primary care physician. You are examining a patient who has come to your primary care clinic. Please respond with the most likely diagnosis and the next two most likely differential diagnoses based on the attached information.”

The scripts were based on the scripts used in previous studies [2] with the consensus of two diagnostic radiologists and one trainee radiologist. Each prompt was submitted to the model only once, and the first response generated was used for evaluation. The accuracy of the primary diagnosis and two differential diagnoses generated by the models were determined by consensus between one trainee radiologist and one board-certified diagnostic radiologist with 11 years of experience. McNemar’s test was used to assess the difference in correct response rates for the overall accuracy between Conditions 1, 2, and 3. Two-sided *p* values < 0.05 were considered statistically significant. Statistical analyses were performed using R (version 4.1.1; R Foundation for Statistical Computing, Vienna, Austria).

## Results

**Fig. 1:**
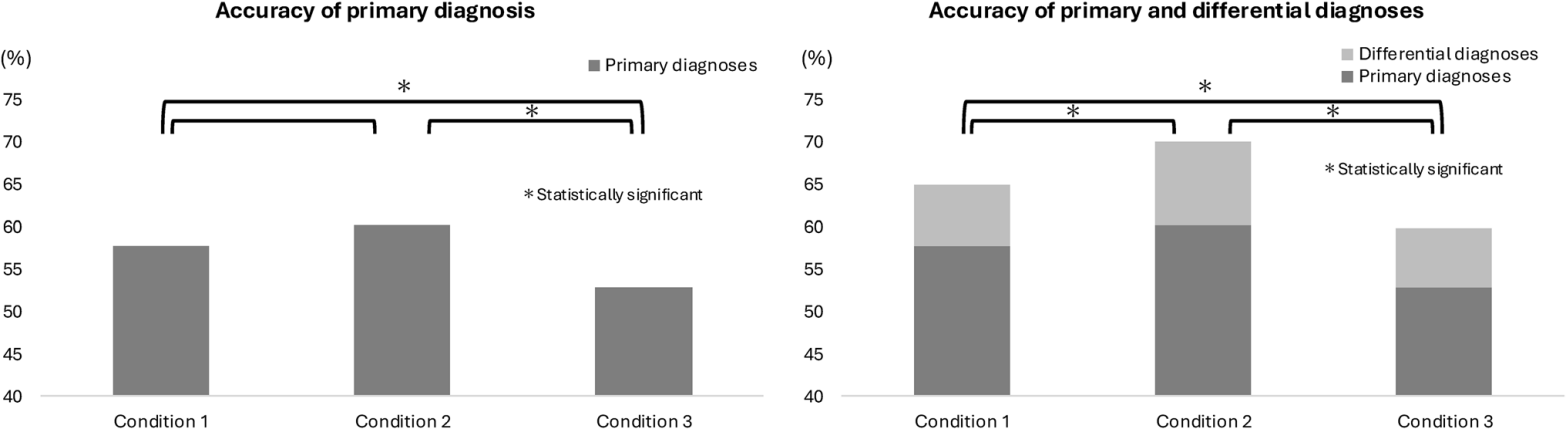
Accuracy of primary diagnosis (left) and combined primary and differential diagnoses (right) significantly increased in Condition 2 and significantly decreased in Condition 3

The overall accuracy rate was significantly improved in Condition 2 compared with Condition 1 (226/322 (70.2%) vs. 209/322 (64.9%), p = 0.029). On the other hand, the overall accuracy rate was significantly decreased in Condition 3 compared with Condition 1 (193/322 (59.9%) vs. 209/322 (64.9%), *p* = 0.027) (Fig. 1 and Table 1).

**Table 1:** Results

	Accuracy			McNemar’s test			
	Condition 1	Condition 2	Condition 3	Condition 2 vs. 1	Condition 2 vs. 3	Condition 3 vs. 1	Condition 3 vs. 2
Primary	186/322 (57.8%)	194/322 (60.2%)	170/322 (52.8%)	*p* = 0.29	*p* < 0.001*	*p* = 0.032*	*p* < 0.001*
Differential diagnoses	23/322 (7.1%)	32/322 (9.9%)	23/322 (7.1%)				
Sum	209/322 (64.9%)	226/322 (70.2%)	193/322 (59.9%)	*p* = 0.029*	*p* < 0.001*	*p* = 0.027*	*p* < 0.001*

Condition 1: Not given as being a quiz case and no situation is set

Condition 2: Given as being quiz cases

Condition 3: Presented as a primary care situation

*Statistically significant

Examples where listing common diseases in Condition 1 resulted in incorrect answers, but listing rare diseases in Condition 2 led to correct answers are summarized in Table 2 [13, 14].

**Table 2:** Examples where differences between correct and incorrect responses under Conditions 1 and 2

	Correct answer	Incorrect diagnosis under Condition 1	Correct diagnosis under Condition 2	Incorrect diagnosis under Condition 3
Case 85	Pelvic Actinomycosis in Association with an Intrauterine Device	Pelvic Inflammatory Disease (PID) with tubo-ovarian abscess	Actinomycosis	Pelvic inflammatory disease
		Diverticulitis with abscess formation	Diverticulitis	Diverticulitis
		Colorectal cancer with abscess formation	Colorectal cancer	Colorectal cancer
Case 258	Granulomatous Prostatitis	Prostate cancer	Prostate cancer	Prostate cancer
		Prostatitis	Prostate abscess	Benign prostatic hyperplasia
		Metastasis from urothelial carcinoma	Granulomatous prostatitis	Chronic prostatitis

Condition 1: Not given as being a quiz case and no situation is set

Condition 2: Given as being quiz cases

Condition 3: Presented as a primary care situation

Examples where listing uncommon diseases in Condition 1 resulted in correct answers, but listing common diseases in Condition 3 led to incorrect answers are summarized in Table 3 [15, 16].

**Table 3:** Examples where differences between correct and incorrect responses arose under Conditions 1 and 3

	Correct answer	Correct diagnosis under Condition 1	Correct diagnosis under Condition 2	Incorrect diagnosis under Condition 3
Case 181	Synovitis Acne Pustulosis Hyperostosis Osteitis (SAPHO) Syndrome	Metastatic melanoma with bone involvement	SAPHO syndrome	Metastatic melanoma
		Vertebral osteomyelitis/diskitis	Metastatic melanoma	Osteomyelitis
		SAPHO syndrome (Synovitis, Acne, Pustulosis, Hyperostosis, and Osteitis)	Vertebral osteomyelitis	Diskitis
Case 201	Glomus Tumor of the Breast	Glomus Tumor of the Breast	Angiolipoma	angiolipoma
		Angiolipoma	Glomus tumor	fibroadenoma
		Vascular malformation	Hemangioma	breast cancer

Condition 1: Not given as being a quiz case and no situation is set

Condition 2: Given as being quiz cases

Condition 3: Presented as a primary care situation

## Discussion

In this study, we utilized cases from Radiology’s “Diagnosis Please,” a radiological image diagnosis quiz for radiologists. When provided with additional information that may influence prior probabilities, specifically informing Claude 3.5 Sonnet that these were quiz cases, we observed a significant improvement in the diagnostic performance of the LLM (overall accuracy rate increased from 64.9% to 70.2%). Conversely, when given incorrect additional information, such as setting the context as a primary care clinic, a significant decrease in diagnostic performance was observed (overall accuracy rate decreased from 64.9% to 59.9%).

Accurate performance evaluation and understanding of LLM characteristics are crucial for exploring their potential applications. Case collections like “Diagnosis Please,” which provide detailed clinical histories, radiological images, and confirmed final diagnoses that can be logically deduced from the given information, are well-suited for LLM performance evaluation (e.g., comparisons between vendors or versions within the same vendor) and assessing similarities and differences with human radiologists. Various authors have previously conducted such studies [1–6, 11]. However, while previous studies have assigned the role of a radiologist to LLMs, they have not informed the LLM that the cases were from quizzes. We believe this created a non-negligible gap that should be addressed.

Human radiologists can adjust their diagnostic approach based on institutional and regional characteristics. This is based on Bayes’ theorem, which describes the probabilistic nature of clinical reasoning and indicates that accurate recognition of current conditions affecting prior probability is essential for improving diagnostic accuracy [8, 9]. Given this context, we hypothesized that providing LLMs with additional information that may influence the prior probabilities of diseases in the target patient group/cohort would improve their diagnostic performance. Furthermore, we posited that providing accurate vs. inaccurate additional information would lead to variations in diagnostic performance for the same set of cases.

The results supported our hypothesis. In Condition 2, where we provided prompts that aligned the assumed prior probabilities with the situation, emphasizing rare diseases, the performance improved. In contrast, in Condition 3, where we provided prompts that deviated from this assumption by emphasizing common diseases, the performance declined. These results suggest that providing prompts that may influence prior probabilities produces outcomes analogous to the effects on human clinical reasoning, although the reasoning process of LLMs remains a black box. This finding implies that LLMs may be incorporating Bayesian-like principles in their diagnostic approach. In addition to optimizing the reasoning steps within the LLM by improving the way information is provided, as shown by Sonoda et al. [11], it was also shown that the diagnostic ability of the LLM can be enhanced by teaching the nature of the given information externally through prompt engineering.

It is difficult to properly consider the reasons that led to the present results because the exact mechanism of LLM is not provided by vendors. We feel that similarity with the concept of “data poisoning attack” may be helpful in explaining why setting a false situation of primary care in this study reduced the diagnostic performance of LLM. Data poisoning attack is a technique that induces LLM to generate erroneous information or biased responses by injecting harmful information into the LLM’s training data. It is known that LLM can present false facts when exposed to data poisoning attack [17]. Although this study did not use methods such as data poisoning attack that directly intervene and manipulate the training data, giving different assumptions by prompt engineering is a similar concept. Prompt engineering can be used to set up situations that LLM deviates from previously learned data, which may have led to a reduction in diagnostic performance. This suggests that we should set up situations which avoid circumstances similar to data poisoning attack when LLM performs diagnostic tasks in order to improve diagnosis accuracy. In other words, it is important for better diagnosis to make an effort to collect as much information about prior probability as possible and apply it to prompt engineering. Note that in the field of machine learning, it is known that the performance of a classifier improves when the correct prior probabilities are input [18].

The results of this study suggest several directions for future research. Just as informing the LLM about the quiz nature of the cases improved its diagnostic performance, providing context about real clinical situations may enhance LLM performance in actual clinical settings. For instance, supplying the LLM with information from databases on prevalence rates specific to regions or institutions could optimize its diagnostic results for those particular settings, making it more valuable in our practice. This underscores the growing importance of developing databases for individual regions and institutions. Proper ethical review processes will be essential to enable the input of clinical data into LLMs. In addition, we performed verification with only Claude 3.5 Sonnet model because it showed the best results in a previous report [4], testing the present issue using other LLMs such as GPT-4o and Gemini 1.5 pro would be helpful in understanding whether the present results are specific to the Claude 3.5 Sonnet or whether they are also generally obtained for other LLMs.

This study has several limitations. The analysis was based on a limited number of cases, precluding subgroup analysis by disease categories. Because LLMs do not always return the same response to the same prompt, retests may yield different results. As the answer criteria set by the “Diagnosis Please” creators are not publicly available, our judgments of correct and incorrect answers may differ from the actual standards. Additionally, since all cases have been published as papers, there is a possibility that they were used in training the LLM.

We demonstrated that in radiological image diagnosis quizzes, providing prior information about the quiz nature of the cases significantly improved the diagnostic performance of Claude 3.5 Sonnet. Conversely, giving incorrect context, such as a primary care setting, significantly decreased its performance. Similar to human physicians, the concept of prior probability, as suggested by Bayes’ theorem, appears to be crucial for the LLM. This implies that constructing and providing optimized databases for specific regions and institutions to LLMs could enhance their diagnostic performances, potentially allowing LLMs to contribute more substantially to real clinical practice.

## Abbreviation

LLM: Large language model

## References

[1] Ueda D, Mitsuyama Y, Takita H, Horiuchi D, Walston SL, Tatekawa H, et al. ChatGPT’s diagnostic performance from patient history and imaging findings on the diagnosis please quizzes. Radiology. 2023;308(1): e231040. 37462501 10.1148/radiol.231040

[2] Horiuchi D, Tatekawa H, Shimono T, Walston SL, Takita H, Matsushita S, et al. Accuracy of ChatGPT generated diagnosis from patient’s medical history and imaging findings in neuroradiology cases. Neuroradiology. 2024;66(1):73–9. 37994939 10.1007/s00234-023-03252-4

[3] Kurokawa R, Ohizumi Y, Kanzawa J, Kurokawa M, Sonoda Y, Nakamura Y, et al. Diagnostic performances of Claude 3 Opus and Claude 3.5 Sonnet from patient history and key images in Radiology’s “Diagnosis Please” cases. Jpn J Radiol. 2024;42(12):1399–1402. 39096483 10.1007/s11604-024-01634-z PMC11588754

[4] Sonoda Y, Kurokawa R, Nakamura Y, Kanzawa J, Kurokawa M, Ohizumi Y, et al. Diagnostic performances of GPT-4o, Claude 3 Opus, and Gemini 1.5 Pro in “Diagnosis Please” cases. J Radiol. 2024;42(11):1231–35. 10.1007/s11604-024-01619-y PMC11522128 38954192

[5] Oura T, Tatekawa H, Horiuchi D, Matsushita S, Takita H, Atsukawa N, et al. Diagnostic accuracy of vision-language models on Japanese diagnostic radiology, nuclear medicine, and interventional radiology specialty board examinations. Jpn J Radiol. 2024;42(12):1392–98. 39031270 10.1007/s11604-024-01633-0 PMC11588758

[6] Horiuchi D, Tatekawa H, Oura T, Oue S, Walston SL, Takita H, et al. Comparing the diagnostic performance of GPT-4-based ChatGPT, GPT-4V-based ChatGPT, and radiologists in challenging neuroradiology cases. Clin Neuroradiol. 2024;34(4):779–87. 38806794 10.1007/s00062-024-01426-y

[7] Uy EJB. Key concepts in clinical epidemiology: Estimating pre-test probability. J Clin Epidemiol. 2022;144:198–202. 34740785 10.1016/j.jclinepi.2021.10.022

[8] Bours MJ. Bayes’ rule in diagnosis. J Clin Epidemiol. 2021;131:158–60. 33741123 10.1016/j.jclinepi.2020.12.021

[9] Westbury CF. Bayes’ rule for clinicians: an introduction. Front Psychol. 2010;1:192. 21833252 10.3389/fpsyg.2010.00192 PMC3153801

[10] Gill CJ, Sabin L, Schmid CH. Why clinicians are natural bayesians. BMJ. 2005;330(7499):1080–83. 15879401 10.1136/bmj.330.7499.1080 PMC557240

[11] Sonoda Y, Kurokawa R, Hagiwara A, Asari Y, Fukushima T, Kanzawa J, et al. Structured clinical reasoning prompt enhances LLM’s diagnostic capabilities in diagnosis please quiz cases. Jpn J Radiol. 2024. 10.1007/s11604-024-01712-2. 39625594 10.1007/s11604-024-01712-2 PMC11953165

[12] Brown TB, Mann B, Ryder N, et al. Language models are few-shot learners. Adv Neural Inf Process Syst. 2020;33:1877–901.

[13] Lely RJ, van Es HW. Case 85: pelvic actinomycosis in association with an intrauterine device. Radiology. 2005;236(2):492–4. 16040905 10.1148/radiol.2362031034

[14] de Leon AD, Costa DN, Francis F, et al. Case 258: Granulomatous Prostatitis. Radiology. 2018;289(1):267–71. 30230997 10.1148/radiol.2018161272

[15] Sweeney SA, Kumar VA, Tayar J, et al. Case 181: synovitis acne pustulosis hyperostosis osteitis (SAPHO) syndrome. Radiology. 2012;263(2):613–7. 22517965 10.1148/radiol.12101436

[16] El Khoury M, Tran-Thanh D, Terrone D, et al. Case 201: glomus tumor of the breast. Radiology. 2014;270(1):302–6. 24354380 10.1148/radiol.13120919

[17] Han T, Nebelung S, Khader F, et al. Medical large language models are susceptible to targeted misinformation attacks. NPJ Digit Med. 2024;7:288. 39443664 10.1038/s41746-024-01282-7 PMC11499642

[18] Saerens M, Latinne P, Decaestecker C. Adjusting the outputs of a classifier to new a priori probabilities: a simple procedure. Neural Comput. 2002;14(1):21–41. 11747533 10.1162/089976602753284446

